# Evidence of attentional bias toward body stimuli in men

**DOI:** 10.3758/s13414-022-02466-7

**Published:** 2022-03-30

**Authors:** Daniel Talbot, Daniella Saleme

**Affiliations:** 1 The University of Notre Dame, Australia, Fremantle, Australia; 2 Western Sydney University, Penrith, Australia

**Keywords:** Attentional bias, Men, Review, Eating disorders, Body image

## Abstract

Over the past 30 years, attentional bias for body shape and weight content has been implicated in the precipitation and maintenance of body dissatisfaction and eating disorders. Although the existence of this bias toward body stimuli is well-established in female populations, it is comparatively understudied in men. This review aimed to examine the nature of this visual attentional bias toward male bodies in male samples across a range of different attentional paradigms, including eye-tracking, dot-probe, and the visual search task. Results were heterogenous, finding some evidence that men with higher body dissatisfaction and eating disorder symptoms demonstrated an attentional bias toward desirable bodies of *other* men, and undesirable features of their own bodies. These results suggest that schematic cognitive models of body dissatisfaction and eating disorders body may also be applicable to men, however more research is needed.

## Body dissatisfaction and cognitive models

Body dissatisfaction can be defined as a negative subjective evaluation of one’s body as a whole or relating to specific aspects of one’s body such as body size, shape, muscularity/muscle tone, and weight (Grogan, 2016). Over the past 30 years, schema theory has been integrated into cognitive models of body dissatisfaction and eating disorders. First introduced by Markus et al. (1987), these models propose that maladaptive body self-schemas are key to the etiology and maintenance of eating disorders. Body self-schemata are defined as cognitive generalizations about one’s own body that are drawn from past experiences. These self-schemata guide and organize the processing of body-related information (Markus et al., 1987). Since Markus et al.’ (1987) introduction, self-schema-based cognitive models of body dissatisfaction and eating disorders have been developed and integrated by Cooper (1997, 2005), Vitousek and Hollon (1990), and then later by Williamson and colleagues (Williamson, 1996; Williamson et al., 1999, 2004).

Cooper (1997, 2005) summarized the primary hypotheses derived across cognitive theories of body dissatisfaction and eating disorders that (i) core beliefs will reflect global negative evaluations of the self; (ii) dysfunctional styles of reasoning or information processing errors and biases will be found in food and eating and in weight and shape concerns; (iii) schema driven processes will be evident in areas of core belief concerns, and (iv) early experience will be important in the formation of core beliefs.

Vitousek and Hollon’s (1990) cognitive model of body dissatisfaction and eating disorder assumes that body dissatisfaction and eating disorder symptoms are developed and maintained by information processing biases stemming from existing body weight and shape-related schema. For individuals with high levels of body dissatisfaction and eating disorder symptoms, these processing biases function to automatically direct attention toward schema-congruent body information (such as a fat fold on one’s own body or thin bodies in the environment) and away from schema incongruent information (such as positive features of one’s own body; Vitousek & Hollon, 1990).

**Fig. 1:**
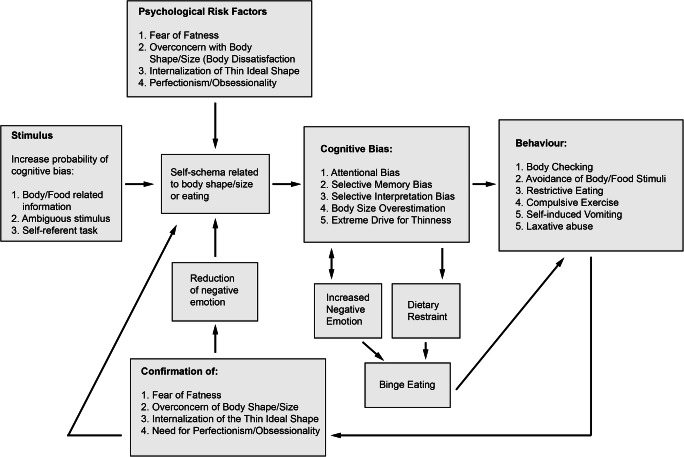
Williamson and colleagues’ cognitive-behavioral model of body dissatisfaction and eating disorders. Adapted from Williamson et al. (2004)

Williamson et al. (2004) built upon perspectives of cognitive and behavioral theorists and schema theory to propose a cognitive-behavioral model of body dissatisfaction and eating disorders (Fig. 1). This model presents the most recent development of the cognitive-behavioral model, and postulates that psychological risk factors such as (i) persistent body dissatisfaction/over-concern with body shape and weight, (ii) fear of fatness, (iii) internalization of the thin ideal body, and (iv) perfectionistic/obsessive traits can result in a negative body self-schema that is reactive to external cues. This self-schema directs an individual’s attention toward body-related stimuli. For example, an individual may pay more attention to their perceived “unattractive” features of their own body, compared with other areas of their body. Conversely, they may be drawn to attractive features of others’ bodies. Additionally, this self-schema guides a negative interpretation bias of self-relevant events. These events can be directly related to body information or can be ambiguous or self-relevant tasks (tasks that require an individual to self-reflect or self-assess in some way). Williamson et al. (2004) provide the example of feelings of being full being interpreted as “feeling fat.” These schematic influences help to provide “evidence” for, and thus reinforce negative assumptions and beliefs about one’s own body (i.e., negative body self-schema congruent assumptions and beliefs). The model posits that these cognitions are beyond conscious awareness and that the person experiences these cognitions as reality.

## Attentional bias

Attentional bias can be defined as the tendency of one’s perception to be affected by recurring, salient thoughts (Bar-Haim et al., 2007). For example, various studies have demonstrated attentional bias toward threatening or phobic-related stimuli in individuals with anxiety disorders (Bar-Haim et al., 2007; Mogg & Bradley, 2005; Mogg et al., 1995). The concept of attentional bias has been implicated as a core factor in all three schematic cognitive models of body dissatisfaction and eating disorders discussed above (Cooper, 1997, 2005; Vitousek & Hollon, 1990; Williamson et al., 2004). These models theorize that underlying schemata produce an attentional drive toward schema-congruent body weight and shape-related information. In this way, negative body-related self-schemata may be the cause of these biases, and in turn, attentional biases may help maintain and reinforce negative body-related self-schema. Due to this cyclical relationship, the existence and role of attentional biases toward body-shape and weight-related stimuli have been identified as a key maintenance factor of body image issues and eating disorders (Cass et al., 2020; Williamson et al., 2004). The role of attentional bias as a maintenance factor is an important consideration as the primary treatments for body-image-related disorders are, for many, ineffective (Downs & Blow, 2013; Hay, 2013; Wilson et al., 2007). The existence and understanding of attentional biases in those with body-image-related disorders could lead to and inform new treatments, such as attentional bias modification (ABM) programs, in which individuals are trained to attend away from stimuli that reinforces negative body-related self-schema in order to weaken it (Renwick et al., 2013). ABM programs have already shown to be effective in reducing symptoms in individuals with anxiety disorder (Beard et al., 2012; Waters et al., 2014) and have also been argued to be applicable to individuals with body-image-related disorders (Renwick et al., 2013). However, the specific relationship between attentional bias and different types of body-image-related stimuli, and therefore attention’s role in the etiology and maintenance of eating disorders, is still in the process of being solidified. Thus far, there have been a large number of studies that have examined this relationship in samples of women with eating disorders, and nonclinical women. Although there is some inconsistency across studies, generally there is moderate evidence that, in line with what is posited in cognitive models, women with high body dissatisfaction tend to display attentional biases toward desirable body features of others, and features of their own body that are perceived as undesirable, compared with women with low body dissatisfaction (Jiang & Vartanian, 2018; Kerr-Gaffney et al., 2019; Starzomska, 2017). Although current evidence does not conclusively support the application of cognitive models to attentional biases in men, the limited evidence does suggest further investigation is warranted.

## Attentional bias and body dissatisfaction in male samples

As stated above, the relationship between attentional bias toward body (weight and shape) stimuli and body dissatisfaction in women has been examined in many studies through a variety of paradigms, however very few have dealt with men. This may be problematic as body weight and shape concerns, and indeed psychopathology of male eating disorders may differ from females (Darcy et al., 2012; Gorrell & Murray, 2019). This difference is largely determined by the difference in body ideals between the sexes: women tend to idealize a thinner body, whilst men strive for a lean and muscular body (Ridgeway & Tylka, 2005). This has significant implications for the cognitive models of eating disorders discussed above. For instance, Gorrell and Murray (2019) highlight that overvaluation of weight and shape is predicated on the thin (female) ideal. Further, dietary restriction present in eating disorders such as anorexia nervosa is likely motivated by a drive to attain respective ideals: for women, it is to attain a slim body; for men, it may be to sufficiently expose and accentuate muscle shape and tone (Gorrell & Murray, 2019).

However, a recent systematic review conducted Rodgers and DuBois (2016) asserts that the role of attention in confirming negative body self-schemata within cognitive models of eating disorders might be equivalent for men and women. They concluded that across the sexes, individuals with high body dissatisfaction may be engaging in a “self-deprecating” bias, attending to desirable features of bodies in the environment and undesirable features of their own bodies, and thus confirming their beliefs about the inadequacy of their own bodies. The authors also assert the role of appearance comparison and rumination in contributing to this process (Rodgers & DuBois, 2016). Based on evidence from female samples (Jiang & Vartanian, 2018; Kerr-Gaffney et al., 2019; Starzomska, 2017) and Rodgers and DuBois’s (2016) review, it is expected that men with greater levels of body dissatisfaction and eating disorder psychopathology would demonstrate attentional biases toward schema-congruent stimuli (i.e., more desirable body features of others’ bodies, and less desirable features relating to one’s own body).

## Empirical Evidence of male attentional bias

When evaluating the empirical evidence for attentional bias toward bodies in cognitive models of eating disorders for men, the results are somewhat mixed. Results are discussed below in relation to (1) examining the evidence for attentional biases toward desirable male bodies of others and (2) toward undesirable features of one’s own body across a range of different paradigms for measuring attention. Results are summarized in Table 1.

**Table 1:** Summary of studies examining attentional bias toward body stimuli in men

Study	Sample Size	Sample Type	Mean age (*SD*)	Methodology	Description of Attentional Relationship
Cho and Lee (2013)	45	South Korean university students	22.20 (2.58)	Eye-tracking	High body dissatisfaction, greater gaze duration and fixation frequency toward desirable male mesomorph bodies.
Cordes et al. (2016)	45	German weight-training men	28.7 (11.70)	Eye-tracking	High drive for thinness, longer gaze duration on features of own body that they were least satisfied with, and shorter gaze duration on features of own body that they were most satisfied with.
Dondzilo et al. (2021a, b)	70	Australian university students	N/A	ARDPEI task	Heightened attention to muscular bodies associated with appearance comparisons, which were linked to greater eating-disorder rumination and greater body dissatisfaction.
Jin et al. (2018)	108 (54 low MD risk; 54 high MD risk)	Chinese gym attendees	N/A	Dot Probe	High risk of muscle dysmorphia, shorter reaction time, shorter saccade latency, and longer fixation duration for images of hypermuscular bodybuilders.
Joseph et al. (2016)	69	U.S. university students	21.60 (5.30)	Dot Probe	High body dissatisfaction, attentional orientation bias for low body fat bodies.
Nikkelen et al. (2012)	50	Dutch university students	23.10 (2.70)	Eye-tracking	After exposure to ideal male bodies: low body dissatisfaction, greater fixation on ideal abdominal body regions. After exposure to neutral stimuli: High body dissatisfaction, greater fixation on ideal abdominal body regions.
Porras-Garcia et al. (2020)	40	Spanish university students	24.75 (2.96)	Eye-tracking	Men with high muscle dissatisfaction had greater gaze duration and a greater number of fixations on muscular areas of interest.
Stephen et al. (2018)	32	Australian university students and community sample	20.85 (3.85)	Eye-tracking	High body dissatisfaction, higher number and greater duration of fixations to thin male bodies.
Talbot et al. (2019)	63	Australian university students	21.91 (4.92)	Visual Search Task	High muscle dissatisfaction, eating restraint, and shape concern, faster reaction time searching for muscular body targets. High eating restraint, faster reaction time searching for obese body targets.
Waldorf et al. (2019)	72 (24 MD; 24 WT; 24 NWT)	German men from the community with MD, weight training, and non-weight training	MD = 23.88 (3.27) WT = 23.00 (3.26) NWT = 24.92 (4.26)	Eye-tracking	Men with MD and nonweight training men had longer gaze duration toward ‘negative’ areas of their own body. Men with MD displayed longer gaze toward hypermuscular bodies.
Warschburger et al. (2015)	24 (12 OW; 12 HW)	German university students and members of obesity support group	OW = 27.67 (9.09) HW = 25.50 (2.84)	Eye-tracking	Obese men held their gaze for longer periods on attractive regions of their own body and others’ bodies, compared with unattractive body regions.

*Note*: OW = overweight or obese; HW = healthy weight; MD = muscle dysmorphia; WT = weight training; NWT = non-weight training

**Bias toward desirable body features of others.** Nine studies were identified showing that men who are dissatisfied with their bodies tend to display attentional biases toward desirable body features of others. Using eye tracking, Stephen and colleagues (2018) found that men with higher body dissatisfaction directed a greater number of fixations and greater total gaze duration to thin male bodies compared with men with lower body dissatisfaction. Similarly, Cho and Lee (2013) found that men with higher body dissatisfaction displayed greater gaze duration and fixation frequency toward desirable male mesomorph bodies compared with men with lower body dissatisfaction. Nikkelen et al. (2012) showed that men who were exposed to neutral images, and then a desirable male body tended display greater number of fixations and greater dwell time on male abdominal regions (i.e., the male six-pack) if they were dissatisfied with their body, compared with those with low body dissatisfaction. Waldorf et al. (2019) found that men diagnosed with muscle dysmorphia displayed longer gaze duration when presented with images of hypermuscular bodies, compared with weight training and nonweight training men. Further, Warschburger et al. (2015) found that obese men display longer total fixation duration on nominated attractive regions of others’ bodies compared with unattractive regions.

Jin et al. (2018) evaluated attention toward male bodies using a visual probe task. They found that men at higher risk of muscle dysmorphia displayed a shorter reaction time, shorter saccade latency, and longer fixation duration for images of hypermuscular bodybuilders, compared with images of bodybuilders with smaller musculatures. Additionally, they found that men at higher risk of muscle dysmorphia displayed faster orientation and longer gaze duration toward muscular bodies compared with low-risk men. Also using a visual dot probe, Joseph et al. (2016) found that, after controlling for body-mass index, men with high body dissatisfaction displayed attentional orientation biases (evidenced through faster reaction time in locating the probe) for low-body-fat bodies, compared with men with low body dissatisfaction.

Talbot et al. (2019) utilized a visual search task to examine attentional orientation bias toward muscular and obese bodies in men. In trials in which desirable muscular bodies were the target stimuli, men with greater muscle dissatisfaction, eating restraint, and body shape concern were faster to find the target compared with those with lower muscle dissatisfaction, eating restraint, and body-shape concern. Effectively, these studies taken together suggest that men with greater body dissatisfaction tend to display faster attentional orientation, greater gaze duration, and greater fixation frequency toward desirable male mesomorph bodies (i.e., bodies of lower body-fat percentage and greater muscularity).

More recently, Dondzilo et al. (2021b) investigated the potential mediating role of appearance comparisons utilizing an Attentional Response to Distal vs. Proximal Emotional Information (ARDPEI) task. This task allowed for measures of attentional engagement and disengagement with desirable body stimuli compared with neutral body stimuli (i.e., a non-mesomorph male body). Self-report body dissatisfaction, eating disorder rumination, and appearance comparison was also recorded. Results indicated that heightened attention to muscular bodies was associated with appearance comparisons, which were linked to greater eating-disorder rumination and as a result, greater body dissatisfaction (Dondzilo et al., 2021b).

### Bias toward undesirable features of one’s own body

Only three studies were identified that evidenced an attentional bias directed toward undesirable features of one’s own body in those with greater body dissatisfaction. Using eye-tracking, Waldorf et al. (2019) found that men diagnosed with muscle dysmorphia and a non-weight-training control group both demonstrated longer gaze duration toward areas of their own body which they perceived as negative compared with areas of their own body that they perceived as attractive. Of note, the attentional bias exhibited by the non-weight training control group is counter to what would be expected based on cognitive models of body dissatisfaction and eating disorders. Waldorf et al. (2019) account for this result as a potential artifact in the procedure: the trait measures of the control group were taken before the experimental aspect of the procedure. During the experimental aspect of the procedure, all participants were photographed wearing minimal clothing under studio lights, and this process may have triggered body dissatisfaction (and therefore attentional bias) in the nonmuscular control group to a greater extent than the weight-training groups before attentional biases were measured.

Similarly, Cordes et al. (2016) found that when presented with images of their own body, weight training men with a high drive for thinness displayed longer gaze duration on features of their body that they were least satisfied with, and shorter gaze duration on features of their body that they were most satisfied with compared with men with low drive for thinness. Porras-Garcia et al. (2020) presented a virtual avatar of male participants’ own bodies in virtual reality, and simultaneously measured gaze duration and frequency through eye tracking. They found that men with high muscle dissatisfaction displayed greater gaze duration and a greater number of fixations on muscular areas of interest (i.e., the chest and shoulders) compared with men with low muscle dissatisfaction. Although they did not ask participants to identify undesirable areas of their own body, the authors suggest that these features may be particularly salient for men with high muscle dissatisfaction as they represent areas in which they would like to improve (Porras-Garcia et al., 2020).

### Evidence inconsistent with proposed cognitive models

As stated above, it was expected that men with greater levels of body dissatisfaction and eating disorder psychopathology would demonstrate attentional biases toward desirable features of others’ bodies, and undesirable features of one’s own body. However, not all studies identified in the present review found results that fall in line with what was expected. Notably, Nikkelen et al. (2012) found that men with low body dissatisfaction who were exposed to ideal male bodies in media demonstrated greater dwell time and number of fixations on ideal body parts (e.g., muscular abdomen) of other men, compared with men with high body dissatisfaction. The authors offer that this attentional pattern might be explained inspiration for self-improvement to increase masculinity, that is those who viewed ideal bodies may have thought of how they might improve their own bodies, resulting in an increase in body satisfaction (Nikkelen et al., 2012). Also converse to expectations, Cordes et al. (2016) found that men with high drive for muscularity displayed longer gaze duration toward attractive parts of their own body, compared with men with lower drive for muscularity. However, after alpha adjustment, these results were not significant.

Talbot et al. (2019) found that men who had higher eating restraint—a behavior symptomatic of some eating disorders—demonstrated an attentional bias for obese bodies (i.e., faster reaction times when searching for an obese target, compared with when searching for a neutral target). This again is counter to what would be expected—that men with greater eating disorder psychopathology would attend to ideal features of another, not “undesirable” features. In their study, the authors conceptualized an obese body as a feared, undesirable stimulus for men (Talbot et al., 2019), suggesting that an evolutionary perspective of adaptive cognitive biases for feared, dangerous objects may account for this attentional pattern (Gilbert, 1998). In this way, it might be that a bias driven by a fear of being fat—a bias also likely related to a negative self-body schema—overrides avoidance of undesirable features of another’s body.

Further, Warschburger et al. (2015) found that obese men held their gaze for longer periods on attractive regions of their own body (and control bodies, as stated above) compared with unattractive body regions. The authors account for this by suggesting that this fixation on the attractive self might be an avoidance behavior to reduce participants’ discomfort. Indeed, avoidance behavior is another maintained factor implicated in cognitive models of eating disorders (Williamson et al., 2004). Notably, Warschburger et al. (2015) examined attentional bias via fixation duration, meaning that it is possible that participants with obesity may have initially orientated toward unattractive regions, before averting their gaze or ‘avoiding’ the distressing stimuli.

## Attentional trends across results

Taking these results into consideration, two trends in line with the nature of attentional biases asserted by Rodgers and DuBois (2016) emerge: (1) body dissatisfied men appear to have attentional biases toward *another’s* ideal body (i.e., a body with a high level of muscularity and low body fat), and (2) dissatisfied men tend to have attentional biases toward perceived negative or nonideal features of their own body. However, there are at least four studies which provide evidence counter to this proposed attentional model. So, what does this mean for schematic cognitive models of body dissatisfaction and eating disorders? Effectively, these results suggest some evidence that existing cognitive models (Cooper, 1997, 2005, Vitousek & Hollon, 1990; Williamson et al., 2004) could be applicable to men, meaning that attentional bias toward body stimuli may be a good candidate factor in the precipitation and perpetuation of body dissatisfaction and eating disorders. Thus, attentional biases toward bodies may be an important factor to consider in formulation and diagnosis of men with body-image-related disorders such as anorexia nervosa and muscle dysmorphia, and present potential new avenues for treatment such as ABM programs (Renwick et al., 2013). However, the relationship may be more complex for both men and women than these cognitive models suggest. Recently, through their proposed serial mediation model of attentional engagement, Dondzilo et al. (2021a) implicated factors such as appearance comparisons and rumination as integral in the relationship between attentional biases and body dissatisfaction and eating disorders. Thus, the lack of consideration of these variables across most of these reviewed studies could account for their inconsistency. Ultimately, additional research with consideration of social comparison and rumination is required for more conclusive support.

## Limitations and directions for future research

One important limitation of the extant research emphasized by these three studies, but also present in all studies included in this review, is the inconsistency in methodology and how attentional bias is conceptualized. Studies included in this review returned four distinct paradigms, eye tracking, the dot probe task, ARDPEI task, and the visual search task, that were used to measure attentional patterns. One might expect a different *kind* of attentional process depending on what was required for each respective task. For instance, some eye-tracking tasks require participants to gaze freely at a single image, whilst a visual search may task the participant with responding as rapidly as possible to each experimental trial. Within paradigms, there proved to be further variation, such as the variation in how attentional bias is conceptualized between eye-tracking studies (e.g., initial fixation, number of fixations, frequency of fixations, duration of fixation). A further example is seen in that typical measures of attention such as the dot probe task are unable to differentiate between attentional engagement and attentional disengagement biases, and as such are measuring different facets of attentional bias (Dondzilo et al., 2021b). As such, identification of the specific mechanisms underpinning body dissatisfaction and methods of measuring, such mechanisms is crucial. Additionally, reported limitations relating to the reliability of paradigms such as the dot-probe paradigm or eye tracking (Schmukle, 2005; Skinner et al., 2018; Staugaard, 2009) should be further explored and remedied if possible.

A further limitation of the existing examination of attentional bias in men is the sheer lack of empirical data. All existing studies identified for this review, of which there were only 11, returned a combined sample of 618 men (*M* = 54.8), a relatively low number compared with the magnitude of research that has been conducted on their female counterparts and hardly large enough to conclusively generalize the nature of these biases to the male population. Additionally, many of these participants were nonclinical and/or university students (approximately 65%). Of the remaining 35% of participants, 108 (18%) were Chinese gym attendees (Jin et al., 2018), 69 (11%) were German weight-training men pooled across two studies (Cordes et al., 2016; Waldorf et al., 2019), 24 (4%) were German men with muscle dysmorphia (Waldorf et al., 2019), and 12 (2%) were overweight or obese men from an obesity support group (Warschburger et al., 2015).

A final limitation of the extant literature is that to date, there are no studies investigating whether body-related attentional biases play a causal role in body image and eating disturbances in men. Causality could sufficiently be examined through the use of ABM paradigms (Renwick et al., 2013). For instance, using ABM to induce attentional bias toward desirable characteristics of another’s body and undesirable characteristic of one’s own body in order to observe whether participants’ body dissatisfaction increases as a result.

Based on these limitations, several recommendations for future studies are proposed. First, future studies should determine the ideal paradigm for best examining attentional bias toward body stimuli. Although on face-value eye-tracking may present as the most comprehensive measure of attentional patterns, the field would benefit from an experimental comparison of the sensitivity for detecting attentional bias across the four paradigms. Notably, paradigms that provide measures of attentional engagement and attentional disengagement, such as the ARDPEI, should be utilized (Dondzilo et al., 2021b). Second, future researchers should strive for clarity, accuracy, and consistency within experimental design and reporting results in terms conceptualizing the nature of their chosen index of attentional bias (i.e., reaction time, initial fixation, number of fixations, frequency of fixations, duration of fixation). Third, replicating existing studies with larger and more diverse samples of men is recommended. This would help consolidate the nature of attentional biases toward differing body types (i.e., muscular, lean, obese) and further our understanding of the applicability of schematic cognitive models of body dissatisfaction and eating disorders to men. Fourth, integration of additional mediating factors (such as social comparison and rumination, as proposed by Dondzilo et al., 2021a) should be considered when examining the relationship between attentional biases toward body stimuli and body dissatisfaction. Fifth, attentional biases (and their magnitude) should be examined in clinical male populations such as men with eating disorders and body dysmorphia. Sixth, the causal nature of attentional biases toward body stimuli should be examined through use of ABM paradigms. Last, should the causal relationship between attentional biases for body stimuli and eating disorder psychopathology can be sufficiently evidenced, it would then be appropriate to evaluate the effectiveness of ABM programs designed to attenuate eating disorder symptoms.
